# Quality of life in patients who undergo conventional or robotic-assisted total laparoscopic hysterectomy: Protocol for a systematic review of randomized controlled trials

**DOI:** 10.1097/MD.0000000000015974

**Published:** 2019-06-07

**Authors:** Rodrigo Corvino Rodrigues, Meline Rossetto Kron Rodrigues, Noélle de Oliveira Freitas, Marilza Vieira Cunha Rudge, Silvana Andréa Molina Lima

**Affiliations:** a Postgraduate Program in Clinical Research; b Department of Nursing, São Paulo State University (UNESP), Medical School, Botucatu; c Postgraduate Program in Nursing, University of Guarulhos (UNG); d Department of Gynecology, Obstetrics and Mastology, São Paulo State University (UNESP), Medical School, Botucatu, São Paulo, Brazil.

**Keywords:** hysterectomy, laparoscopy, quality of life, robotics, systematic review

## Abstract

**Background:**

Hysterectomy for benign gynecologic diseases, especially dysfunctional uterine bleeding, is one of the most common gynecologic interventions. The uterus can be removed using abdominal, vaginal, laparoscopic, or robotic-assisted laparoscopic hysterectomy. In a robotic-assisted procedure, the surgeon directs the robot while seated at a console in the operating room. This differs from laparoscopic hysterectomy because a “robot” performs the operation, while the surgeon watches a monitor. This systematic review will compare quality of life (QOL) in patients who undergo total robotic-assisted laparoscopic hysterectomy for benign indications and those who undergo conventional laparoscopic surgery.

**Methods:**

We will perform a systematic review according to the Cochrane Methodology for randomized controlled trials. The review will include studies reporting use of QOL metrics to assess patients who undergo total hysterectomy for benign indications using robotic-assisted technique or conventional laparoscopic surgery. QOL will be the primary outcome and will be measured using validated instruments. An overall search strategy will be developed and adapted for Embase, MEDLINE, LILACS, and CENTRAL databases. Two reviewers will independently select the eligible studies, assess the risk of bias, and extract the data from included studies. Similar outcomes measured in at least 2 trials will be plotted in the meta-analysis using Review Manager 5.3. The quality of evidence will be determined using the GRADE approach.

**Results:**

This systematic review is designed to provide high quality evidence on QOL in patients undergoing total hysterectomy for benign indications using either robotic-assisted or conventional laparoscopic surgery.

**Conclusion:**

It is expected that high-quality evidence on QOL can be used to guide decision-making by institutions and clinicians to improve health care; the evidence can also be used in future studies.

**PROSPERO registration number:**

PROSPERO CRD 42019129913

## 1 Introduction

Technologic advances have led to increasing use of robots for repetitive tasks that demand high accuracy.^[1,2]^

In the health care field, robots were initially introduced to assist in basic tasks, but are now used in surgical procedures.^[3]^ The robotic evolution in surgery began with the introduction of NeuroMate, which was developed for use in stereotactic brain biopsy and approved by the Food and Drug Administration in 1999.^[4]^

Robotic-assisted surgery has been beneficial for both patients and surgeons, as the technique offers 3-dimensional vision with zoom capability, increased surgical dexterity, minimization of movement, the possibility of teleoperation, elimination of hand tremor, and better ergonomics.^[5]^ Robotic-assisted surgery has also been associated with lower risk of bleeding and infection and has led to reduced postoperative pain scores, with less trauma and faster recovery. The repeatability, stability, accuracy, millimeter-scale dexterity, and use of multiple monitoring sensors are also cited as benefits of the technique.^[6]^

Robots are now routinely used in head and neck, gastrointestinal, gynecologic, cardiac, and urologic procedures.^[5,6]^ Surgical procedures can result in complications, prolonged hospitalization, need for recurrent surgery, and difficulty in performing daily postoperative activities, and can be associated with self-image disorders, impaired self-esteem, and depression.^[6,7]^

The presence of postoperative complications can compromise quality of life (QOL), limit performance of physical, work, and domestic activities, and negatively affect emotional and personal relationships.^[7]^ QOL is defined as “the individual's perception of their position in life in the context of the culture and value system in which they live and in relation to their goals, expectations, standards and concerns,” according to the World Health Organization.^[7]^

As use of robots has become widespread, especially in health care and surgery, the evaluation of QOL in patients who undergo robotic-assisted surgery is of great relevance, in light of the reportedly good outcomes, lower risk of infection, reduced blood loss and operative time, and faster postoperative recovery.^[8]^

The only surgical treatment option previously available to women with abnormal vaginal bleeding has been hysterectomy. Currently, benign diseases such as fibroids, adenomyosis, and endometrial polyps can undergo hormonal treatment, but many gynecologic diseases still require surgery.^[9]^

Hysterectomy for benign gynecologic disease, especially for dysfunctional uterine bleeding, uterine prolapse, or uterine fibroids, is one of the most common gynecologic interventions, accounting for about 600,000 procedures per year in the United States.^[10]^ It is estimated that about 30% of 60-year-old women have already undergone hysterectomy, with 590,000 hysterectomies performed each year in the United States. Removal of the uterus can be performed in several ways. Abdominal hysterectomy (known as laparotomy) involves removal of the uterus through a low abdominal incision.^[11]^ Vaginal hysterectomy involves removal of the uterus through the vagina without an abdominal incision. Laparoscopic hysterectomy is performed through small incisions in the abdomen, with the uterus removed through the vagina or one of the small abdominal incisions after morcellation (fragmentation). Different types of laparoscopic hysterectomy are performed, depending on the extent of surgery required. Robotic-assisted laparoscopic hysterectomy has been recently introduced.^[12]^ In a robotic-assisted procedure, the surgeon directs the robot while seated at a console in the operating room. This differs from laparoscopic hysterectomy because the “robot” performs the procedure, while the surgeon watches a monitor. To make well-informed decisions, women who need to undergo hysterectomy for benign disease need to know the risks and benefits of each surgical approach.^[11,12]^

A retrospective cohort study found that patients undergoing robotic-assisted laparoscopic hysterectomy have a significantly lower risk of readmission <30 days after surgery, compared to those who undergo conventional laparoscopic, abdominal (open), or vaginal hysterectomy. Patients in the robotic-assisted cohort also had shorter inpatient stays, less estimated blood loss, and reduced costs associated with readmission when compared to those who underwent non-robotic-assisted approaches. Prospective records describing quality outcomes, total costs including 30 days of follow-up, and QOL should be reviewed to confirm these findings and determine which surgical route offers the greatest value to the patient and society.^[13]^

A systematic review with inclusion of 4 randomized controlled trials (RCTs) aimed at assessing the safety and efficacy of robotic vs laparoscopic hysterectomy in women with benign uterine disease found no significant difference in the rate of postoperative complications. The study reported that costs, pain scores, and QOL assessments were not available for analysis and that the role of robotic-assisted surgery in benign gynecologic disease remained unclear.^[14]^ Thus, the objective of this systematic review is to assess and compare QOL in patients who undergo total hysterectomy for benign indications using robotic-assisted technique or conventional laparoscopic surgery.

## 2 Methods

### 2.1 Study registration

This systematic review will be conducted according to the Cochrane Collaboration,^[15]^ and reported according to the PRISMA statement.^[16]^ The protocol for this systematic review has been registered in PROSPERO 2019 under number CRD 42019129913.

### 2.2 Eligibility criteria

The study will include RCTs reporting QOL metrics for patients who underwent total hysterectomy for benign indications using robotic-assisted technique or conventional laparoscopic surgery. The reports of interest will assess QOL in patients who underwent total hysterectomy for benign indications using robotic-assisted technique or conventional laparoscopic surgery. The study population will include patients who underwent total hysterectomy for benign indications. The intervention will be robotic-assisted laparoscopic surgery. The comparator will be conventional laparoscopic surgery. The outcomes will be determined using QOL metrics.

#### 2.2.1 Types of participants

This study will include patients who underwent total hysterectomy for benign indications using robotic-assisted laparoscopic surgery or conventional laparoscopic surgery, with assessment of postoperative QOL.

#### 2.2.2 Types of interventions

The intervention of interest will assess QOL in patients who underwent total hysterectomy for benign indications using robotic-assisted laparoscopic surgery.

#### 2.2.3 Comparison

The comparative QOL will be determined in patients who underwent total hysterectomy for benign indications using conventional laparoscopic surgery.

#### 2.2.4 Exclusion criteria

Observational studies, studies with lack of randomization between groups or lack of a control group, and studies with abdominal or vaginal hysterectomy as the control group will be excluded. The outcomes will be determined for QOL assessed with validated instruments. Studies that did not involve humans will be excluded.

#### 2.2.5 Types of outcome measures

The QOL will be the primary outcome, and will be assessed with validated instruments that report metrics in patients who underwent total hysterectomy for benign indications using robotic-assisted technique or conventional laparoscopic surgery.

### 2.3 Search methods for identification of studies

We will consult 4 electronic databases: MEDLINE (PubMed, 1966–2019), Embase (Elsevier, 1980-2019), LILACS (Virtual Health Library, 1982–2019), and CENTRAL (the Registry of Controlled Clinical Studies of the Cochrane Collaboration, 1972–2019). A basic strategy was developed for the search in PubMed and adapted for other electronic databases. They search will be conducted using Descriptors in Health Sciences and Medical Subject Headings (MeSH). The descriptors include “Robotics,” “Hysterectomy,” and “Quality of Life.” The basic search strategy will be (with PubMed as an example):

- #1 “Quality of Life” [MeSH] OR Life Quality OR Health-Related Quality Of Life OR Health Related Quality Of Life OR HRQOL
- #2 “Robotics” [MeSH] OR Remote Operations (Robotics) OR Operation, Remote (Robotics) OR Operations, Remote (Robotics) OR Remote Operation (Robotics) OR Telerobotics OR Soft Robotics OR Robotic, Soft OR Robotics, Soft OR Soft Robotic
- #3 “Hysterectomy” [MeSH] OR Hysterectomies
- #1 AND #2 AND #3

#### 2.3.1 Searching other resources

There will be no language restriction, but only human studies will be selected. References to selected articles, including review articles, will be assessed to identify all relevant studies. Reference manuals of clinical papers will be searched in relevant journals. Any related conference abstracts, and unpublished or ongoing trials will also be included. The Clinical Trials website (https://clinicaltrials.gov/) and the Brazilian ReBEC Clinical Trials website (http://www.ensaiosclinicos.gov.br/) will be consulted for possible ongoing studies.

### 2.4 Study selection

**Figure 1:**
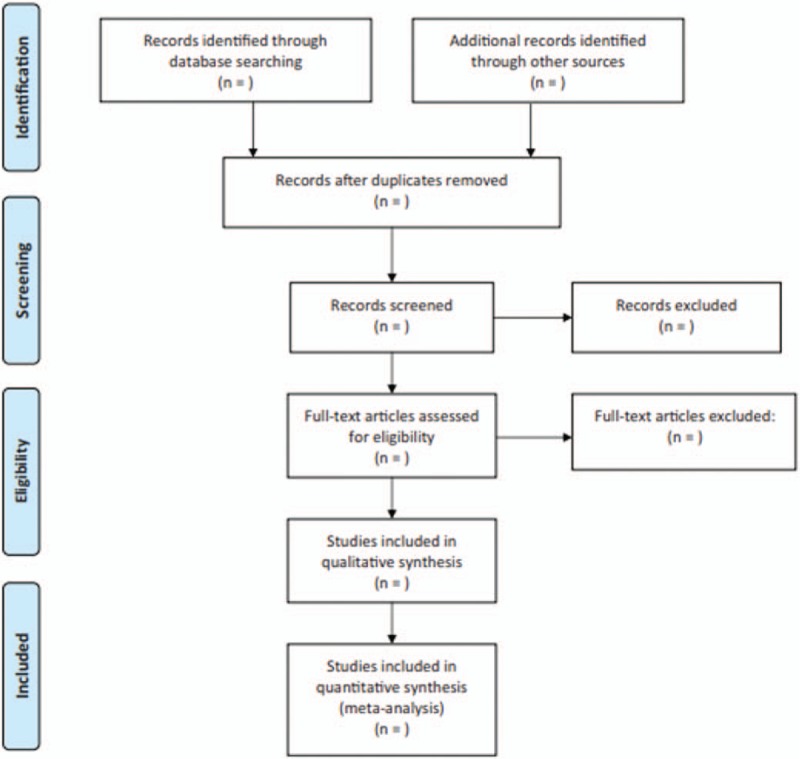
Flow diagram of selected studies.

Two researchers will perform all study selection independently. Any conflict regarding the study selection between 2 researchers will be solved by a 3rd researcher through discussion. At the 1st stage, researchers will review the titles and abstracts of all records to identify any potential studies based on the predefined eligibility criteria. At the 2nd stage, full texts of all potentially relevant studies will be read for further selection. The entire study selection procedure will abide by the PRISMA guidelines, and will be shown in a PRISMA flow chart (Fig. 1).

### 2.5 Data extraction and management

Two researchers will independently extract the relevant data from each full-text article using a standardized form based on the Cochrane Handbook,^[17]^ with the following information: study characteristics (design, method of randomization), participants, interventions, measurement of QOL, scores, instrument used, and analysis performed. The selection will be reviewed for accuracy, and any discrepancies will be resolved by consensus or discussion with another researcher. In case of duplicate publications or more reports from the primary study, data extraction will be optimized using the best available information for all items from the same study.

### 2.6 Risk of bias (quality) assessment

Two sets of researchers will independently evaluate the risk of bias in each eligible RCT. Any discrepancies will be resolved by consensus or discussion with another investigator. The Cochrane Collaboration tool will be used to assess risk of bias in RCTs.^[18]^ The following items will be assessed: generation of allocation sequence (selection bias), concealment of allocation sequence (selection bias), blinding (detection and performance bias), blinding of participants and staff for evaluation of results, incomplete data results (attrition bias), reports with selective results (information bias), and other biases. For each RCT, each item will be described as having a low risk of bias, a high risk of bias, or a clear risk of bias.

### 2.7 Quality of evidence rating

The Grading of Recommendations Assessment, Development and Evaluation (GRADE) tool will be used to evaluate the overall strength of the evidence.^[19]^ The results will be summarized in tables as a summary of findings. limitations of the study, inconsistencies, indirect evidence, inaccuracies, and publication bias will be considered. Evidence quality will be classified into 4 levels: high, moderate, low, or very low.

### 2.8 Strategy for data synthesis, assessment of heterogeneity, and subgroup analysis

If possible, this systematic review will perform a meta-analysis using fixed- and random-effects models (when necessary) and the Mantel–Haenszel method. Associations will be reported as relative risk with 95% confidence interval (CI). Heterogeneity will be assessed with the Cochran Chi-squared test, and the degree of heterogeneity will be quantified with the *I*^2^ statistic and 95% CI. An *I*^2^ value between 30% and 60% will be described as moderate heterogeneity. Publication bias will be evaluated with funnel plots and formally assessed with the Egger test. For variability in results between studies, the *I*^2^ statistic and the *P*-value obtained from the Chi-squared Cochrane test will be used. Review Manager software (RevMan; version 5.3; Nordic Cochrane Center, Cochrane) will be used for all analyses.^[20]^ If necessary, the meta-analysis will be performed as a subgroup analysis based on the type of instrument used to measure QOL. If meta-analysis is not possible, the results will be compiled and presented in tabular form.

### 2.9 Ethics and dissemination

As no primary data collection will be undertaken, ethics approval is not required. We plan to present the findings of this systematic review in a peer-reviewed scientific journal. We also intend to present preliminary and completed findings at appropriate conferences.

## 3 Discussion

A previous study on this subject was published in 2016; however, the present review is based on incremental data. Moreover, evidence is still lacking in the literature.^[16]^ The previous systematic review and meta-analysis had an umbrella effect, in which the main outcome was confirmation of safety and efficacy of robotic vs laparoscopic hysterectomy in women with benign uterine disease. The present review aims to identify the primary outcome of QOL in these patients, in addition to following the Cochrane methodology and using the GRADE method to evaluate the strength of evidence. The expectation is that the solid data and robust evidence obtained will be used in clinical practice or future studies, as well as by institutions that offer these procedures.

## Acknowledgment

The authors thank Editage to review the language of our manuscript.

## Author contributions

**Conceptualization:** Rodrigo Corvino Rodrigues, Meline Rossetto Kron Rodrigues, Silvana Andréa Molina Lima.

**Formal analysis:** Rodrigo Corvino Rodrigues, Meline Rossetto Kron Rodrigues, Noélle de Oliveira Freitas.

**Funding acquisition:** Meline Rossetto Kron Rodrigues, Noélle de Oliveira Freitas.

**Investigation:** Rodrigo Corvino Rodrigues, Noélle de Oliveira Freitas.

**Methodology:** Rodrigo Corvino Rodrigues, Meline Rossetto Kron Rodrigues, Noélle de Oliveira Freitas, Marilza Vieira Cunha Rudge.

**Supervision:** Meline Rossetto Kron Rodrigues, Marilza Vieira Cunha Rudge, Silvana Andréa Molina Lima.

**Validation:** Meline Rossetto Kron Rodrigues, Marilza Vieira Cunha Rudge, Silvana Andréa Molina Lima.

**Visualization:** Rodrigo Corvino Rodrigues, Silvana Andréa Molina Lima.

**Writing – original draft:** Rodrigo Corvino Rodrigues, Meline Rossetto Kron Rodrigues, Noélle de Oliveira Freitas, Marilza Vieira Cunha Rudge, Silvana Andréa Molina Lima.

**Writing – review & editing:** Rodrigo Corvino Rodrigues, Meline Rossetto Kron Rodrigues, Noélle de Oliveira Freitas, Silvana Andréa Molina Lima.

Meline Rossetto Kron Rodrigues orcid: 0000-0003-2174-268X.
